# Bacteriums and Typhoid Fever

**Published:** 1864-02

**Authors:** 


Bacteriums and Typhoid Fever.—Prof. Sigri has called the attention of the French Academy to the presence of these infusoria in the blood of a man who died of typhoid fever in the hospital of Sienna.—*Lancet, Jan.* 2, 1864.

